# Epidemiology of Pediatric Astrovirus Gastroenteritis in a Nicaraguan Birth Cohort

**DOI:** 10.1093/ofid/ofae465

**Published:** 2024-08-16

**Authors:** Rebecca J Rubinstein, Lester Gutiérrez, Christian Toval-Ruíz, Kelli Hammond, Lars Bode, Jan Vinjé, Samuel Vilchez, Sylvia Becker-Dreps, Filemón Bucardo, Nadja A Vielot, Yaoska Reyes

**Affiliations:** Department of Epidemiology, University of North Carolina at Chapel Hill, Chapel Hill, North Carolina, USA; Centro de Investigación en Enfermedades Tropicales, Facultad de Microbiología, Universidad de Costa Rica, San José, Costa Rica; Facultad de Ingeniería,Universidad Tecnológica La Salle, León, Nicaragua; Department of Family Medicine, University of North Carolina at Chapel Hill, Chapel Hill, North Carolina, USA; Department of Pediatrics, Larsson-Rosenquist Foundation Mother-Milk-Infant Center of Research Excellence, Human Milk Institute, University of California San Diego, La Jolla, California, USA; Division of Viral Diseases, Centers for Disease Control and Prevention, Atlanta, Georgia, USA; Department of Family Medicine, University of North Carolina at Chapel Hill, Chapel Hill, North Carolina, USA; Department of Epidemiology, University of North Carolina at Chapel Hill, Chapel Hill, North Carolina, USA; Department of Family Medicine, University of North Carolina at Chapel Hill, Chapel Hill, North Carolina, USA; Department of Family Medicine, University of North Carolina at Chapel Hill, Chapel Hill, North Carolina, USA; Department of Microbiology and Immunology, University of North Carolina at Chapel Hill, Chapel Hill, North Carolina, USA; Department of Family Medicine, University of North Carolina at Chapel Hill, Chapel Hill, North Carolina, USA; Department of Epidemiology, University of North Carolina at Chapel Hill, Chapel Hill, North Carolina, USA

**Keywords:** astrovirus, birth cohort, children, diarrhea, Nicaragua

## Abstract

**Background:**

Astrovirus is a leading cause of acute gastroenteritis in children worldwide. However, few prospective studies have analyzed astrovirus in community-dwelling pediatric populations in low- and middle-income countries.

**Methods:**

We assessed the incidence, risk factors, clinical characteristics, genotypes, viral coinfections, and time distribution of astrovirus gastroenteritis in 443 healthy Nicaraguan children born in 2017 to 2018 who were followed for 36 months. Children were recruited from hospitals and birth records in an economically diverse neighborhood of León city. Astrovirus-positive episodes and genotypes were identified from stool with reverse transcription quantitative polymerase chain reaction and Sanger sequencing.

**Results:**

Of 1708 total specimens tested, 80 children (18%) experienced at least 1 astrovirus episode, and 9 experienced repeat episodes, mostly during the rainy season (May–October). Initial astrovirus episodes were not associated with a lowered risk against future episodes. In exploratory analyses, home toilets were associated with a lower risk of future astrovirus episodes (hazard ratio, 0.19; 95% CI, .04–.91). Human astrovirus 5 episodes, representing 15% of all typed episodes, were associated with longer diarrhea and more symptomatic rotavirus coinfections.

**Conclusions:**

Astrovirus was a common cause of gastroenteritis in this cohort, and future studies should clarify the role of astrovirus genotype in clinical infection severity.

Human astroviruses are an important cause of acute gastroenteritis (AGE) worldwide, detected in 2% to 9% of all AGE cases in children aged <3 years [1]. Infections are typically self-limiting with 1 to 4 days of watery diarrhea, fever, and abdominal pain [2] and, rarely, extragastrointestinal infection and death in adults and children who are immunocompromised [3]. Astrovirus frequently appears as a coinfection with other enteric viral pathogens, especially rotavirus, norovirus, and sapovirus [1]. Coinfections with other pathogens have been associated with more severe gastroenteritis symptoms and greater likelihood of experiencing diarrhea in low- and middle-income country (LMIC) settings [1, 4], where incidence, morbidity, and mortality from childhood gastroenteritis are highest.

Astroviruses are nonenveloped single-stranded positive-sense RNA viruses in the family Astroviridae. Enteric infections are primarily caused by human astrovirus (HAstV) types 1 to 8 in the *Mamastrovirus* genus, MAstV-1 species [5]. Like other RNA viruses, astrovirus has a high rate of evolution and recombination [6]. Consequently, additional astroviruses, including the Melbourne and Virginia types, were discovered with improved sequencing technology. Unlike HAstV genotypes 1 to 8, the Melbourne and Virginia astroviruses are more closely related to animal astroviruses and more commonly associated with extragastrointestinal infections in humans, especially in neurologic cases.

With few exceptions, most astrovirus studies in LMICs have been conducted in health care settings, limited to children with more severe clinical symptoms [1, 4, 7–9]. These studies could potentially overestimate typical symptom duration in astrovirus infection. In addition, children presenting to health care settings may be wealthier, which could bias estimates of risk factors for infections, as such children do not represent the population as a whole, although this bias may be less pronounced in countries with universal health care, including Nicaragua. Cross-sectional studies of hospitalized children are also susceptible to recall bias if caregivers misreport the timing of risk factors and clinical symptoms. Community-based cohort studies may better represent the populations at risk for astrovirus and capture the full spectrum of disease severity and risk factors. However, investigations of astrovirus in prospective birth cohorts in Central America have not been conducted in >20 years [4, 8].

In a prospective cohort study with weekly follow-up over 3 years, we describe the incidence and potential risk factors for astrovirus AGE in Nicaraguan children.

## METHODS

### Study Sample

The Sapovirus-Associated Gastro-Enteritis study is a population-based birth cohort of 443 mother-infant dyads in León, Nicaragua, born between 12 June 2017 and 31 July 2018 and followed until infants were 36 months old [10]. Children were recruited from maternity hospitals and birth registries in an economically diverse neighborhood of León, the second-largest city in Nicaragua. Within 10 to 14 days of birth, field staff recorded the sociodemographic characteristics of the infants (eg, sex, birth history, breastfeeding) and households (eg, floor construction, water source, sanitation). For 36 months, children were surveilled weekly in their households for AGE symptoms of vomiting and diarrhea, where acute diarrhea was defined as an increase in stool frequency to 3 stools per 24-hour period or a substantial change in stool consistency, such as bloody, very loose, or watery stool. A new AGE episode was defined as symptoms preceded by 3 days of no symptoms [10]. At each study visit, mothers were asked if they had breastfed the child on the previous day and whether the child had consumed anything besides breastmilk in the past week. At monthly visits and when an AGE episode was reported, mothers were asked if their children had consumed uncooked fruits or vegetables, seafood, or other foods outside the home within the previous 7 days and whether the children had attended social gatherings or had contact with anyone experiencing diarrhea or vomiting in or outside the home within the previous 7 days [10]. All participants’ families provided informed consent for study participation and biobanking of samples for future research. All study materials were approved by the Ethical Committee for Biomedical Research at the Universidad Nacional Autónoma de Nicaragua–León (Acta 2-2017), the Institutional Review Board at the University of North Carolina at Chapel Hill (protocol 16-2079), and the Centers for Disease Control and Prevention in Atlanta (project 0900f3eb81c526a7) [10, 11].

### Astrovirus Detection and Genotyping

When AGE was reported, a stool specimen was collected within 2 hours of defecation. Stool specimens were transported in a sterile plastic container or soiled diaper at 4 °C to the Microbiology Department of the Universidad Nacional Autónoma de Nicaragua–León and suspended in phosphate-buffered saline as previously described [10]. RNA was extracted from stool suspension via the QIAamp Viral RNA Mini Kit (Qiagen). Astrovirus-specific real-time reverse transcription quantitative polymerase chain reaction (RT-qPCR) on the capsid gene was performed in a 96-well reaction plate with the LightCycler 96 System (Roche) with primers described by Liu and colleagues. RT-qPCR was performed under the following conditions: 45 °C for 10 minutes, 95 °C for 10 minutes, followed by 40 cycles of 95 °C for 15 seconds and 60 °C for 30 seconds as described previously [12]. Samples with cycle threshold values ≤38 were considered positive for astrovirus, and the AGE episode during which the stool was collected was defined as an “astrovirus episode.” All astrovirus-positive stool specimens were then reamplified by conventional RT-PCR; positive samples with cycle threshold values ≤35 were Sanger sequenced; and sequences were genotyped by comparing with astrovirus reference genotypes [13].

### Detection of Viral Coinfections in Stool

RNA from norovirus GI, norovirus GII, sapovirus, and rotavirus was detected via RT-qPCR as described previously [12].

### Human Milk Oligosaccharide Identification

Manually expelled human milk was analyzed for human milk oligosaccharides (HMOs) once from lactating mothers at approximately 2 months postpartum (range, 1–4 months). Nineteen unique HMOs were extracted, identified, and quantified with fluorescent high- and ultra-high-pressure liquid chromatography developed by the Bode Laboratory at the University of California, San Diego [14]. This method uses the mass concentration and retention time of HMO standards to distinguish among structural isomers, and it quantifies ∼95% of all HMOs by concentration in human milk [14].

### Statistical Analysis

To analyze the molecular epidemiology and frequency of astrovirus in our cohort, we generated a monthly epidemic curve of astrovirus episodes and an epidemic curve by genotype.

We compared potential risk factors and exposures for astrovirus AGE among children who did and did not develop astrovirus AGE (ie, cases and noncases) using a Mantel-Haenszel chi-square test for frequency and percentage and a 2-sample *t* test for mean and SD. Exclusive breastfeeding was defined as the total number of weeks in which the mother breastfed the child, during which the child had not yet consumed other foods or liquids. Any breastfeeding was defined as the total number of weeks in which the mother breastfed the child, regardless of whether the child consumed other foods or liquids.

We determined the relative hazard (95% CI) of children experiencing an astrovirus episode by risk factors of interest identified from a review of the literature and from associations with risk of infection with other pathogens as determined in prior analyses from this cohort. We used Cox models to assess the bivariate associations of baseline covariates as well as time-varying risk factors reported in each week of observation (eg, breastfeeding in the last week) with the incidence of an astrovirus episode [15]. The reported values for time-varying risk factors that were reported on a monthly basis (eg, interpersonal contacts and food consumption) were carried backward for each of the preceding 3 weeks. We also compared clinical characteristics among different astrovirus genotypes using a nonparametric Kruskal-Wallis test for continuous variables and a Fisher exact test of goodness of fit for all categorical variables.

Next, we compared the clinical and epidemiologic characteristics of individuals who developed multiple astrovirus episodes vs 1 episode. Gastroenteritis severity was assessed by a severity score described previously [10, 16] that incorporates symptoms and their duration, as well as receipt of intravenous fluids. The frequencies (percentages) of categorical variables and medians (IQRs) of continuous variables were compared. We also assessed the conditional relative hazard (95% CI) of a second astrovirus episode among children who experienced an initial astrovirus episode, using a Prentice-Williams-Peterson model [17]. Alpha was set at .05 for all inferential tests, with no adjustment for multiple comparisons.

## RESULTS

Among 1708 diarrheal specimens tested from 443 children, 80 children (18%) experienced at least 1 astrovirus AGE episode, and 9 children experienced multiple episodes. The children contributed 12 083 person-months of follow-up, and the incidence rate of astrovirus-specific AGE was 7.9 per 100 child-years (95% CI, 6.2–9.8). The median age of astrovirus episode onset was 16 months (IQR, 9–23). Of the 1710 AGE episodes occurring during follow-up, astrovirus was detectable in 89 (5.2%).

**Figure 1.**
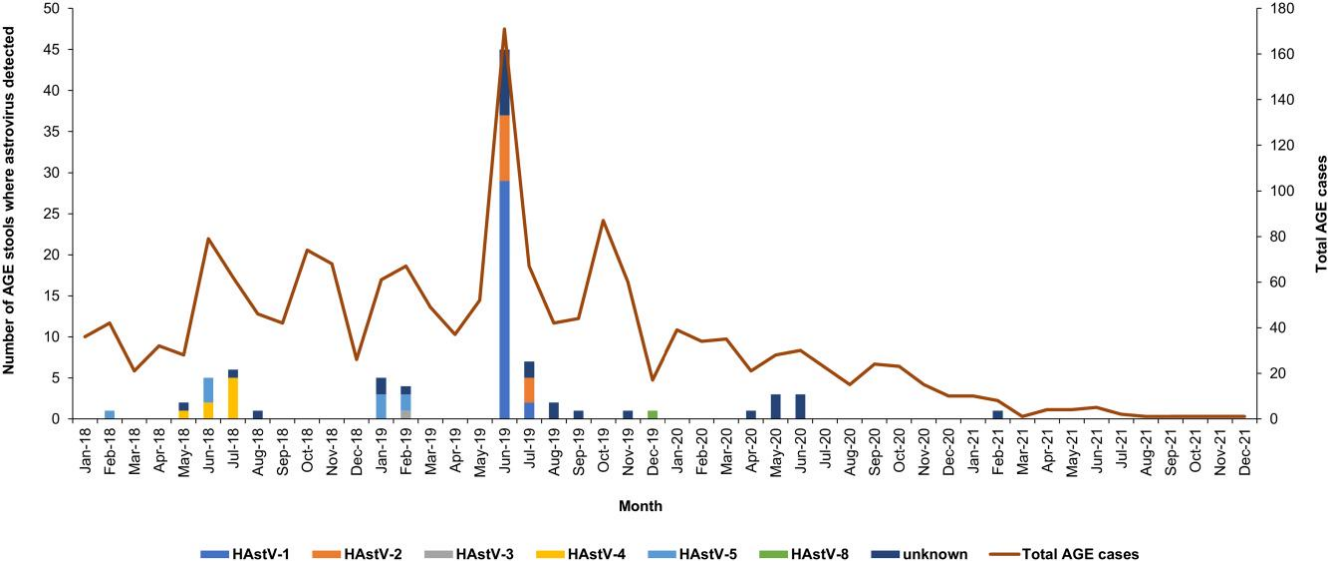
Monthly frequency of astrovirus AGE stratified by genotype as compared with monthly frequency of all AGE in a Nicaraguan birth cohort. AGE, acute gastroenteritis; HAstV, human astrovirus.

The majority of astrovirus episodes occurred during the rainy season (May–October) in the Pacific coastal region of Nicaragua [18], where León is located (Figure 1). No cases were documented during the start (April, May) or end (November) of the rainy season (Figure 1). The largest cluster of episodes occurred between June and September 2019, with 45 episodes detected in June alone. The median age of episode onset among children infected in June or July 2019 was 18 months (IQR, 16–20), slightly older than children infected at other time points (14 months [9–22]).

The infecting genotype was determined in 61 of 89 astrovirus specimens. HAstV-1 was detected in 31 (51%) episodes (Figure 1). Other genotypes included HAstV-2 (18%), HAstV-4 (13%), and HAstV-5 (15%). Only 1 episode of HAstV-3 and HAstV-8 was identified (1.6%). HAstV-4 and HAstV-5 were primarily prevalent in June to July 2018 and January to February 2019. In June to July 2019, HAstV-1 and HAstV-2 were primarily detected. Episodes of astroviruses that could not be typed were similarly distributed across all months.

Few characteristics differed between astrovirus cases and noncases (Table 1). Average length of exclusive breastfeeding was <1 month after cohort entry, precluding further analyses of this risk factor. Children with astrovirus were nonexclusively breastfed longer than those without astrovirus (82 vs 69 weeks), though this difference was not statistically significant. Children who had contact with an individual experiencing diarrhea or vomiting in the previous week were more likely to experience an astrovirus AGE episode than those with no such contact (hazard ratio, 9.08; 95% CI, 5.77–14.29; Table 2).

**Table 1.** Baseline Characteristics of Astrovirus AGE Relative to Noncases in a Birth Cohort in León, Nicaragua

Characteristic	Astrovirus AGE (n = 80)	Noncases (n = 363)	*P* Value ^a^
Birth and genetic characteristics			
Female sex, No. (%)	33 (41)	184 (51)	.1
Mean birthweight in grams (2 missing)	3148 (460.8)	3123 (438.7)	.7
Mean age of mother years at child's birth	23.2 (5.1)	23.8 (5.3)	.3
Born by vaginal delivery, No. (%)	43 (54)	199 (55)	.9
Socioeconomic indicators and household sanitation, No. (%)			
Mother completed any secondary or higher education (12 missing)	63 (79)	273 (75)	.4
Mother employed at time of child's birth	13 (16)	60 (17)	.8
Presence of a toilet in the home	62 (78)	261 (72)	.3
Presence of a dirt floor in the home	27 (34)	107 (29)	.5
Piped municipal water in the home	68 (85)	304 (84)	.8
Nutrition factors			
Any breastfeeding, wk	82 (58.6)	69.0 (58.2)	.07
HMO concentrations, ^b^ μg/mL			
2_FL	3059.9 (1644.3)	2848.9 (1685.7)	.7
3FL	508.4 (663.3)	526.9 (475.3)	.9
DFLac	338.8 (346.1)	322.2 (314.9)	.9
3_SL	153.3 (115.3)	163.4 (148.4)	.8
6_SL	607.8 (294.8)	554.5 (271.4)	.6
LNT	749.2 (494.6)	761.0 (524.2)	.9
LNnT	171.2 (89.0)	171.7 (95.7)	>.99
LNFP_I	1354.7 (902.1)	1114.6 (801.3)	.4
LNFP_II	672.1 (495.0)	706.6 (478.3)	.8
LNFP_III	65.6 (63.6)	59.2 (46.1)	.8
LSTb	117.5 (85.8)	104.9 (64.6)	.6
LSTc	362.9 (212.8)	320.6 (198.4)	.6
DFLNT	1056.5 (809.8)	992.7 (809.2)	.8
LNH	172.5 (96.3)	175.1 (111.8)	.9
DSLNT	228.7 (152.2)	206.9 (127.8)	.6
FLNH	298.3 (151.4)	329.1 (202.2)	.6
DFLNH	121.5 (123.4)	115.5 (97.0)	.9
FDSLNH	250.2 (220.4)	284.9 (232.0)	.6
DSLNH	282.0 (169.0)	259.2 (148.9)	.6

Data are presented as mean (SD) unless noted otherwise.

Abbreviations: AGE, acute gastroenteritis; HMO, human milk oligosaccharide.

^a^Mantel-Haenszel chi-square test for categorical variables; Student *t* test for continuous variables.

^b^A false discovery rate of 0.05 was applied to all comparisons with HMOs.

**Table 2.** Correlates of Symptomatic Astrovirus-Positive Gastroenteritis in a Nicaraguan Birth Cohort (N = 443)

Characteristic	Hazard Ratio (95% CI)
Birth characteristics	
Female sex	0.69 (.45–1.07)
Vaginal delivery	1.02 (.66–1.58)
Mean age of mother at child's birth, y	0.99 (.95–1.04)
Socioeconomic indicators and household sanitation	
Mother completed any secondary education or more	1.22 (.69–2.18)
Mother employed at time of child's birth	0.86 (.61–1.19)
Crowding in the home, >2.5 people/bedroom	0.85 (.52–1.39)
Presence of a dirt floor in the home	1.22 (.77–1.94)
Presence of an indoor toilet in the home	1.23 (.74–2.05)
Piped municipal water in the home	1.15 (.62–2.13)
Experienced a water source interruption in the past week	0.82 (.42–1.60)
Characteristics within the past week ^a^	
Animals present in the home (any)	0.93 (.58–1.48)
Dog	0.90 (.58–1.39)
Cat	1.25 (.78–1.99)
Chickens	0.70 (.41–1.21)
Pig	0.15 (.02–1.09)
Bird	0.91 (.44–1.90)
Other, including horses and/or ducks	0.84 (.37–1.93)
Nutrition	
Breastfed	1.01 (.96–1.07)
Received foods or liquids other than human milk	1.00 (.89–1.13)
Ate uncooked fruit/vegetable	0.71 (.29–1.76)
Ate seafood	1.00 (.93–1.08)
Ate outside the home	1.01 (.96–1.07)
Interpersonal contact	
Attended a social event	1.00 (.93–1.09)
Attended childcare	1.04 (1.02–1.06)
Met/played with a child outside the home	0.99 (.98–1.00)
Used public transportation	1.01 (.96–1.07)
Shared a bottle or cup with another person	0.98 (.83–1.16)
Went swimming	0.86 (.27–2.73)
Had contact with any person with diarrhea and/or vomiting	9.14 (5.80–14.40)

^a^All characteristics described “in the past week” were measured and updated weekly. These exposures were treated as time varying in Cox modeling.

We analyzed clinical characteristics and risk factors among nonastrovirus AGE episodes, initial astrovirus episodes, and subsequent astrovirus episodes (Table 3). Diarrhea was more common in initial astrovirus episodes (100%) than subsequent astrovirus episodes (67%) and nonastrovirus AGE episodes (95%). Conversely, fever was more prevalent in subsequent vs initial astrovirus episodes (49% vs 56%), while fever was less common still in nonastrovirus AGE episodes (39%). Nevertheless, health care utilization was more common among subsequent astrovirus episodes as compared with initial astrovirus episodes and nonastrovirus episodes. Additionally, children were more than twice as likely to receive zinc in a repeat astrovirus AGE episode when compared with an initial episode (67% vs 24%). Coinfection incidence was similar among initial and subsequent cases, except for sapovirus, which was more common among subsequent astrovirus episodes (22% vs 13%). Last, the results of the Prentice-Williams-Peterson model to assess potential risk factors associated with secondary astrovirus episodes showed that while having a toilet in the home was associated with a lower risk of future astrovirus AGE episodes (hazard ratio, 0.19; 95% CI, .04–.91), other maternal and household characteristics were not associated with the risk of secondary astrovirus episodes (Supplementary Table 1).

**Table 3.** Clinical Characteristics of Astrovirus-Positive Gastroenteritis Episodes in a Birth Cohort in León, Nicaragua

	No. (%) or Median (IQR)		
Clinical Characteristic	Nonastrovirus AGE ^a^ (n = 1367)	First Episodes (n = 80)	Subsequent Episodes ^b^ (n = 9)
Diarrhea	1303 (95)	80 (100)	6 (67)
Duration, d	6 (4–9)	6 (3–9)	4 (1–5)
No. of stools in a 24-h period	5 (4–7)	5 (2)	5 (7.5)
Vomiting	352 (26)	24 (30)	3 (33)
Duration, d	2 (2–4)	1 (1–2)	1 (1–3)
Fever	535 (39)	39 (49)	5 (56)
Gastroenteritis severity score, 0–15 ^c^	5 (4–6)	5 (4–7)	5 (2.5–7)
Received care at			
Primary care clinic	479 (35)	19 (24)	3 (33)
Emergency department	151 (11)	4 (5)	1 (8)
Admitted to hospital	59 (4)	2 (3)	1 (8)
Received			
Zinc	423 (31)	19 (24)	6 (67)
Intravenous fluid	52 (4)	1 (1)	1 (8)
Coinfection with other gastrointestinal viruses			
Norovirus GI	NA	13 (16)	1 (8)
Norovirus GII	NA	4 (5)	0
Rotavirus	NA	6 (8)	1 (8)
Sapovirus	NA	10 (13)	2 (22)

Abbreviations: AGE, acute gastroenteritis; NA, not assessed.

^a^Nonastrovirus AGE episodes were defined as episodes of symptomatic AGE where we tested for astrovirus RNA in stool but did not find it.

^b^Eight children experienced a second episode, and 1 child experienced a third.

^c^Severity score calculated by duration of diarrhea and/or vomiting symptoms, maximum number if stools reported per day, presence of fever, and receipt of intravenous fluid for dehydration. Adapted from the scale developed by Lee et al [16].

Repeat episodes from different genotypes were observed in 2 children. One child had HAstV-4 AGE, followed by HAstV-2 AGE over a year later, experiencing diarrhea and vomiting of 5 to 6 days in the HAstV-2 episode but no fever. The other child was infected initially with HAstV-1 and, after 14 symptom-free days, HAstV-2. The HAstV-1 episode was accompanied by diarrhea, vomiting, and fever, while the HAstV-2 episode involved only diarrhea.

Most symptom prevalence, burden, and health care–seeking characteristics did not differ among HAstV-1, HAstV-2, HAstV-4, and HAstV-5 episodes (Table 4). We could not compare characteristics between HAstV-3 and HAstV-8 due to the small numbers of these episodes. Notably, even after extreme outliers in diarrhea duration (25, 88, and 169 days) among HAstV-5 episodes were eliminated, diarrhea lasted significantly longer in HAstV-5 episodes (median, 13.5 days; IQR, 10.5–19.5) than episodes with HAstV-1, HAstV-2, and HAstV-4, where median duration did not exceed 8 days (*P* = .02). Additionally, the maximum diarrhea duration in HAstV-5 episodes (36 days) was >2 times that of all other genotypes (15 days). One-third of all children infected with HAstV-5 were additionally infected with wild type rotavirus, while only 1 additional rotavirus coinfection was identified in a child with HAstV-2 (*P* = .02). The median age at rotavirus/astrovirus coinfections was 14.6 months (IQR, 12.8–16.8). Norovirus GI coinfection was also more common in HAstV-1 (32%) and HAstV-2 (18%) episodes than in HAstV-4 or HAstV-5 episodes (0%), though this difference was not statistically significant (*P* = .07). We found that 8 (26%) of 31 HAstV-1 episodes co-occurred with sapovirus. While duration of diarrhea differed by HAstV strain type, we did not observe differences in median duration of diarrhea by coinfection with sapovirus, norovirus GI/GII, or wild type rotavirus. In fact, episodes with the longest diarrheal duration tended to occur in participants without coinfections, regardless of HAstV strain type.

**Table 4.** Characteristics of Astrovirus-Positive Gastroenteritis Episodes by Astrovirus Genotype in a Nicaraguan Birth Cohort (n = 59)

	No. (%) or Median (IQR)					
Characteristic	HAstV-1 (n = 31)	HAstV-2 (n = 11)	HAstV-4 (n = 8)	HAstV-5 (n = 9)	Test	*P* Value
Diarrhea	31 (100.0)	11 (100.0)	8 (100.0)	9 (100.0)	Fisher exact	>.99
Duration of diarrhea, d	8 (5–10)	5 (3–9)	6.5 (3–8)	15 (11–21)	Kruskal-Wallis	.02
No. of stools in a 24-h period	5 (4–7)	5 (4–6)	5 (4–6)	6 (5–7.5)	Kruskal-Wallis	.53
Vomiting	7 (22.6)	2 (18.2)	3 (37.5)	1 (11.1)	Fisher exact	.83
Duration of vomiting, d	2 (2–3)	3 (3–3)	2 (2–6)	18 (18–18)	Kruskal-Wallis	.21
Fever	16 (51.6)	4 (36.7)	6 (75.0)	3 (33.3)	Fisher exact	.3
Received care at						
Primary care clinic	12 (38.7)	2 (18.2)	2 (25.0)	2 (22.2)	Fisher exact	.63
Emergency department	2 (6.5)	2 (18.2)	1 (12.5)	0 (0.0)	Fisher exact	.42
Admitted to hospital	1 (3.2)	1 (9.1)	0 (0.0)	0 (0.0)	Fisher exact	.73
Received						
Intravenous fluid	0 (0.0)	1 (9.1)	0 (0.0)	0 (0.0)	Fisher exact	.48
Zinc	11 (35.5)	4 (36.4)	5 (62.5)	0 (0.0)	Fisher exact	.09

## DISCUSSION

Few longitudinal birth cohort studies have evaluated the risk of astrovirus in pediatric populations in LMICs [1, 4, 8, 9, 19, 20]. Here, we present the first prospective study of symptomatic astrovirus infection burden in healthy children in Nicaragua and only the second published study on astrovirus infection in Mesoamerica in almost 20 years [4, 7, 8, 19, 21, 22]. Our findings identify potential genotype-specific differences in transmission, symptom duration, and probability of coinfections.

The incidence of astrovirus among AGE episodes (89/1710, 5.2%) was similar to that reported from birth cohorts in Nepal, Bangladesh, India, Pakistan, Peru, Brazil, South Africa, and Tanzania who were followed for 2 years (all 5.6%) [1, 20] and one from Guatemala followed for 3 years (7.3%); it was also lower as compared with a birth cohort from Mexico followed for 3 years (27.0%) and higher than a birth cohort from Egypt followed for 3 years (3.5%) [1, 4, 9, 20]. Notably, our study was conducted almost 20 years after the Egyptian, Mexican, and Guatemalan studies, during which hygiene and sanitation have improved worldwide [23]. Additionally, the molecular testing that we used has higher sensitivity for pathogen detection than the enzyme-linked immunosorbent assays used for antigen detection in the previous studies. Nevertheless, many of our observations corroborate established evidence on astrovirus transmission. As in other tropical settings, most astrovirus episodes occurred during the rainy months of June and July 2019 [4, 8, 24], possibly attributable to poorer sewage control, given astrovirus's fecal-oral route of transmission [3]. This pattern also mirrors sapovirus and norovirus transmission in León, which tends to predominate in the rainy season [25–27]. Children younger than 6 months, many of whom still breastfeed, may be less susceptible to astrovirus than children 12 months and older [1, 28, 29]. Notably, a similar spike in infections did not occur in June and July 2018, when the median age of the cohort was <6 months. Meanwhile, children infected in June and July 2019 had a median age of 18 months, consistent with the epidemiology of astrovirus in other LMIC settings [1, 29].

HAstV-1 was the most common genotype in our cohort, similar to other settings [2, 3, 5, 9, 22]. However, HAstV-1 was not detected during all months of astrovirus transmission, appearing only in June and July 2019. This pattern of detection is consistent with pediatric outbreak investigations [30] and wastewater surveillance [31], in which HAstV-1 is often the only strain detected. Children experiencing AGE during June and July 2019 were slightly older than children infected at other time points. Although children 18 months and older may have a higher risk of astrovirus AGE than younger children [29], the small differences in age among children infected in June and July 2019 as compared with other times do not likely account for the increase in infections during this time.

The only other identified genotype to cocirculate with HAstV-1 was HAstV-2, another commonly detected genotype worldwide [3]. Nonetheless, multiple HAstV genotypes were detected in most months, and unknown genotypes were found in almost every month of transmission. Additionally, HAstV-5 constituted almost 15% of all typed genotypes and 11% of all initial episodes. While HAstV-5 is typically not as widespread as HAstV-1, it was common in studies from Nepal, Egypt, China, and the United States, representing 16% to 42% of astrovirus-positive genotypes [9, 20, 32, 33]. Surprisingly, we found that diarrhea lasted longer in children infected with HAstV-5. Genotype-specific astrovirus infection severity is an understudied area. To our knowledge, only 1 other study analyzed genotype-specific symptom severity and found that HAstV-3 was associated with persistent diarrhea in previously healthy children [34].

We found that coinfections with wild type rotavirus were more common among HAstV-5 episodes than among other genotypes. In Shanghai, China, patients infected with HAstV-1 and HAstV-5 were more likely to be coinfected with another enteric virus than patients infected with HAstV-2, HAstV-3, HAstV-4, or HAstV-8. However, most of these coinfections existed between norovirus and astrovirus [32], unlike our findings. While rotavirus and astrovirus coinfections commonly occur as coinfections in studies of gastroenteritis epidemiology [35, 36], it is not known whether certain HAstV genotypes are more prone to coinfections with rotavirus. Given that astrovirus coinfections with norovirus GI (12 episodes) and sapovirus (10 episodes) were more common overall than astrovirus/rotavirus coinfections and that norovirus and sapovirus most commonly co-occurred with HAstV-1 episodes, our data seem to suggest that different HAstV genotypes may have different propensities for coinfection with other enteric pathogens. This is another poorly understood area that deserves further study [3].

Like Guix and colleagues [37] and Cortez and colleagues [33], we identified isolated cases of repeated astrovirus episodes of different genotypes, suggesting that different astrovirus genotypes may not offer complete cross-protective immunity [3]. Subsequent episodes in the 9 children with >1 astrovirus episode (7 with untyped episodes) were accompanied by fewer and shorter symptoms. However, proportionally more children received zinc, intravenous fluids and hospital care in subsequent rather than initial episodes, conversely suggesting more clinically severe infections. Although our study was strengthened by the large number of children whom we followed for 3 years, we were limited by our inability to screen for asymptomatic astrovirus infections. It is possible that undetected asymptomatic astrovirus exposure early in life protected children from multiple subsequent episodes, limiting our ability to investigate whether astrovirus genotypes offer cross-protection against other genotypes. Future longitudinal studies should consider including astrovirus serology and asymptomatic astrovirus infections to clarify whether astrovirus serotypes offer cross-protection.

Astrovirus is spread through the fecal-oral route among individuals who are infectious [38] and through contaminated food [39], making control and prevention difficult, especially in congregate settings including schools and nursing homes [38]. Although our exploratory analysis of risk factors for astrovirus gastroenteritis identified that baseline food and sanitary factors were not associated with initial astrovirus AGE, we found that having a toilet in the home was associated with a lower risk of a subsequent astrovirus episode.

Strengths of our study include its prospective design, 36-month follow-up with weekly diarrheal surveillance from birth, and recruitment of community-dwelling children. Risk factors collected prospectively, with frequent follow-up, are less likely to be affected by recall bias. Our community-based design is potentially more generalizable and less prone to selection bias and overestimation of astrovirus prevalence and risk factors than hospital- or clinic-based studies. Additionally, we were able identify the genotype for 68% of astrovirus episodes, allowing us to make some comparisons in astrovirus symptom severity or coinfection incidence among serotypes. By directly attempting to genotype all stool associated with AGE cases, we likely achieved greater sensitivity of genotyping than had we initially screened AGE stools with immunoassay and restricted genotyping to positive samples. Unfortunately, we could not type 32% of astrovirus episodes, and we did not assess for coinfections with bacteria or parasites. Thus, it is possible that symptoms observed during a so-called astrovirus episode were actually caused by a different pathogen. With a sample size of 443, we may have been underpowered to detect some differences, although we analyzed a larger sample than other birth cohorts in Central America [4, 19]. Furthermore, the goal of our exploratory epidemiologic analyses was to identify potential risk factors of infection, not to establish definitive causation between any risk factors and astrovirus AGE. A future case-control study with incidence density control sampling, nested within a prospective cohort and with adjustment or weighting for confounders, could address these questions. Last, while our results are likely generalizable to urban settings on the Pacific Coast of Nicaragua, our findings may not explain astrovirus epidemiology in other areas of the country or region.

Our findings provide an estimate of astrovirus AGE risk in a community of healthy children in Central America, where few studies of astrovirus have been conducted. Our data also generate hypotheses about genotype-specific differences in astrovirus natural history and coinfections, as well as the role of HMOs in infection. Larger studies in Central America and other LMIC settings are needed to better understand the impact of astrovirus infections on child health and its prevention in regions with a high burden of diarrheal disease.

## Supplementary Data

Supplementary materials are available at *Open Forum Infectious Diseases* online. Consisting of data provided by the authors to benefit the reader, the posted materials are not copyedited and are the sole responsibility of the authors, so questions or comments should be addressed to the corresponding author.

## Supplementary Material

ofae465_Supplementary_Data

## Notes

***Acknowledgments***. We are very grateful to the parents and children who participated in the Sapovirus-Associated Gastro-Enteritis cohort. This work would not have been possible without our dedicated field and laboratory team members: Fredman González, Merling Balmaceda, Vanessa Bolaños, Nancy Corea, Jhosselyng Delgado, Marvel Fuentes, Yadira Hernandez, Llurvin Madriz, Yuvielka Martinez, Patricia Mendez, Maria Mendoza, Ruth Neira, Xiomara Obando, Yorling Picado, Veronica Pravia, Aura Scott, and Mileydis Soto. We also extend our thanks to the local Ministry of Health office in León and the staff of the Perla Maria Norori Health Center for their support in recruiting study participants. We thank Cloe Sermet, Veronika Sandler, and Nikhila Yerabandi at University of California San Diego for their technical support with HMO analysis. We also thank Mark Sotir for reviewing our manuscript.

***Author contributions***. R. J. R., Y. R., N. A. V., F. B., S. B.-D., J. V., S. V., and C. T.-R. contributed to the conception and design of the study. Y. R., L. G., C. T.-R., L. B., J. V., S. V., F. B., and N. A. V. generated and analyzed the data. N. A. V., K. H., and R. J. R. performed the statistical analysis. R. J. R. wrote the first draft of the manuscript. All authors contributed to the article and approved the submitted version. S. V., L. G., C. T.-R., and Y. R. are former researchers from their cited primary affiliations. Credits are given because the majority of the research was performed while affiliated there.

***Data availability statement***. The raw data supporting the conclusions of this article will be made available by the authors, without undue reservation.

***Patient consent statement***. All participants’ families provided written informed consent for study participation and biobanking of samples for future research. All study materials were approved by the Ethical Committee for Biomedical Research at the Universidad Nacional Autónoma de Nicaragua–León (Acta 2-2017), the Institutional Review Board at the University of North Carolina at Chapel Hill (protocol 16-2079), and the Centers for Disease Control and Prevention in Atlanta (project 0900f3eb81c526a7).

***Financial support*.** This work was supported by the National Institute of Allergy and Infectious Diseases, National Institutes of Health (R01AI127845), to provide funding for the Sapovirus-Associated Gastro-Enteritis study and salary support for S. B.-D., F. B., C. T.-R., L. G., S. V., N. A. V., Y. R.; an international research capacity-building award from the Fogarty International Center, National Institutes of Health (D43TW010923), to provide additional salary support for Y. R., and L. G.; the National Institute of Allergy and Infectious Diseases (K24AI141744 to S. V. and S. B.-D.); the National Institutes of Health (in 2018–2020, T32GM8719; 2022–present, T32DK007634; to R. J. R.); the University of North Carolina Graduate School, GlaxoSmithKline, and CERobs, LLC (R. J. R.); and an endowment from the Family Larsson-Rosenquist Foundation in Switzerland to L. B. as the University of California San Diego Chair of Collaborative Human Milk Research.
