# Supplementary material for: Epidemiology of Pediatric Astrovirus Gastroenteritis in a Nicaraguan Birth Cohort
Source: Open Forum Infect Dis. 2024 Aug 16;11(9):ofae465. doi: 10.1093/ofid/ofae465 (PMC11378399; doi:10.1093/ofid/ofae465)
Supplement: ofae465_Supplementary_Data [file ofae465_supplementary_data.docx]

| **Supplementary Table 1. Risk of subsequent astrovirus AGE among children who experienced 1 or more astrovirus AGE episode*.** | | |
| --- | --- | --- |
| **Maternal characteristic** | **HR (95% CI)** | ***p*** |
| 1-year increase in maternal age at child’s birth | 1.01 (0.87, 1.16) | 0.9 |
| Vaginal v. cesarean birth | 1.55 (0.35, 6.96) | 0.6 |
| Maternal high school education or above (yes/no) | 0.48 (0.09, 2.58) | 0.4 |
| Maternal occupation (formal v. informal sector) | 0.43 (0.07, 2.57) | 0.4 |
| **Household characteristic** |  |  |
| Dirt floor (yes/no) | 1.53 (0.34, 6.94) | 0.6 |
| Access to piped municipal water (yes/no) | 0.46 (0.09, 2.37) | 0.4 |
| Toilet in home | 0.19 (0.04, 0.91) | 0.04 |

*Analyses of risk of recurrent astrovirus AGE episodes with Prentice-Williams-Peterson modeling was limited to time-invariant covariates
